# A Unique Case of Incomplete Bifid Ureter and Associated Arterial Variations

**DOI:** 10.1155/2021/6655813

**Published:** 2021-01-04

**Authors:** Shivika Ahuja, Hannah Sullivan, Mark Noller, Yun Tan, Daniel Daly

**Affiliations:** ^1^Center for Anatomical Science and Education, Department of Surgery, Saint Louis University School of Medicine, Saint Louis, MO 63104, USA; ^2^Urological Surgeons of Northern California, San Jose, CA 95124, USA

## Abstract

*Introduction*. Urogenital and vascular anomalies, including a left duplex kidney and a left aberrant renal artery that gave rise to the left ovarian artery, were observed in a 77-year-old female cadaver during a routine dissection. *Description*. A left aberrant renal artery, which gave rise to the left ovarian artery, was observed originating from the aorta 4 cm below the left renal artery. Two independent contributions to a bifid ureter were found originating from the hilum of the left kidney. These two contributions descended 12.4 cm and 10.6 cm, respectively, posterior to the left aberrant renal artery and lateral to the left ovarian artery before uniting anterior to the psoas major muscle to descend 12.7 cm to the bladder. *Significance*. While the duplex kidney is a relatively common congenital anomaly that can be asymptomatic, it can also potentially be associated with compression of renal vasculature or the ureter. Ureteral compression can then result in several pathologies including reflux, urinary tract infection (UTI), ureteropelvic junction obstruction, or hydronephrosis. Compression of renal and ovarian vasculature can result in altered blood flow to the kidney and ovary, potentially causing fibrosis, atrophy, or organ failure. Current imaging techniques alone are insufficient for correct diagnostics of such complications, and they must be supplemented with a thorough understanding of the respective anatomical variations.

## 1. Introduction

Congenital abnormalities of the kidney and urinary tract (CAKUT) that encompass variations in the kidney, ureter, bladder, or urethra range in severity from asymptomatic to significantly contributing to end-stage renal disease [1]. One of the most common CAKUT variations is the duplex kidney, also known as the duplicated ureteral collecting system [2]. A normal ureter travels 25–30 cm from the kidney to the bladder and is divided into three regions—ureteropelvic junction (UPJ), ureterovesicular junction (UVJ), and the point of crossing over the common iliac arteries [3]. A duplex kidney can exist in one of two anatomical variations: complete and incomplete [4]. Complete duplication results in two patent ureters arising from the affected kidney and draining independently into the bladder, while incomplete duplication results in two ureters draining the same kidney but merging prior to reaching the bladder [5]. The incomplete duplication can be further categorized into either a bifid pelvis, which occurs when the two ureteral contributions unite proximal to the UPJ, or a bifid ureter, in which the ureteral contributions merge distally to the UPJ but proximal to the UVJ [5]. Duplex kidneys are more common in women and are usually observed unilaterally, as was observed in the current case [6, 7]. Imaging techniques alone, such as excretory urography, voiding cystourethrography, and CT without contrast, are not always reliable diagnostic tools for this upper urinary tract abnormality due to the positioning of the ureter. Imaging results must be coupled with thorough knowledge of CAKUT anatomical variations to diagnose possible associated complications, such as various types of ureteral reflux and ureteropelvic junction obstruction (UPJO) [8].

The vascular variation of a left aberrant renal artery giving rise to the left ovarian artery could increase the possibility of urogenital complications resulting from ureteral compression. Current literature suggests that because variation in arterial supply to the kidney is more prevalent than previously believed, there is a need to standardize the nomenclature describing additional renal arteries [9, 10]. Various terms, including accessory, aberrant, anomalous, supernumerary, supplementary, multiple, upper/lower polar arteries, and aortic superior and inferior polar arteries, have been used for that purpose [9]. We will use the definition of an aberrant renal artery established by Holden et al. as an artery which enters the kidney outside of the hilum, whereas an accessory renal artery follows the renal artery to enter the kidney at the hilum; both accessory and aberrant renal arteries typically originate from the abdominal aorta, but this can vary [11].

The current case report is of interest due to its clinical importance and novelty, specifically outlining how this unique combination of urogenital and vascular anomalies can exacerbate their individual complications. Accessory renal arteries have been proposed to independently contribute to hypertension, hydronephrosis, or segmental infarcts while also causing or worsening urogenital symptoms associated with a bifid ureter [12]. Similarly, ureteric obstruction of vascular structures can cause ischemia of their target tissues. This case is educationally and clinically valuable as it emphasizes the importance of foundational knowledge of anatomical variations, which, when used in conjunction with imaging studies, could positively impact patient care.

## 2. Case Presentation

A 77-year-old female body was received through the Saint Louis University Gift of Body Program of the Center for Anatomical Science and Education (CASE) with a signed informed consent form from the donor. The CASE gift body program abides by all the rules set forth by the Uniform Anatomical Gift Act (UAGA).

**Figure 1:**
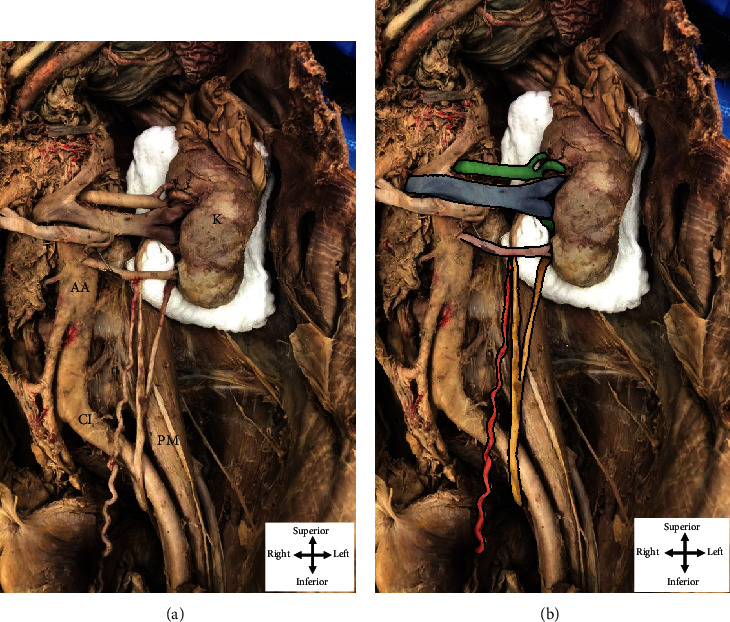
The dissected path of the left bifid ureter and aberrant renal artery. (a) depicts the dissected path of the left bifid ureter with labeling of relevant landmarks while (b) depicts relevant structures pseudocolored for ease in visualization. The left renal vein (blue in (b)) is observed as the anterior-most structure in the renal hilum, thereby serving as a reference point for relationships in this region. The left renal artery (green in (b)), located posterior to the renal vein, has upper two segments that can be observed entering the superior pole of the kidney (K in (a)). The left aberrant renal artery (pink in (b)) is observed originating from the abdominal aorta (AA in (a)) and branching before reaching the inferior pole of the kidney. As the aberrant artery courses toward the left kidney, it gives off the highly coiled left ovarian artery (red in (b)) that descends into the pelvis medial to the left ureter. The superior and inferior contributions to the bifid ureter (yellow) emerge from the posterior aspect of the renal hilum and unite to form the left ureter proximal to the bladder and near the anterior surface of the psoas major muscle (PM in (a)). The left ureter crosses over the left common iliac artery (CI in (a)) before entering the pelvis to join the bladder.

**Figure 2:**
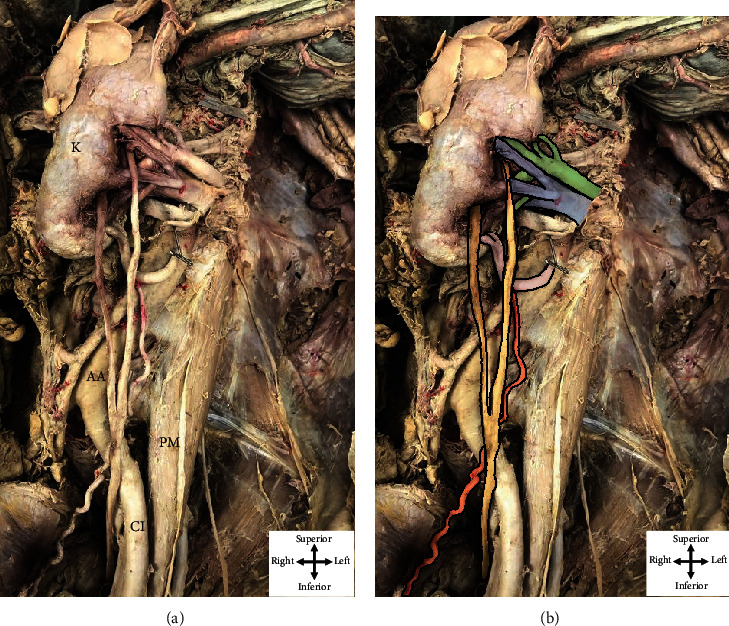
The anatomical presentation of the posterior aspect of the left kidney. (a) depicts the relevant dissected structures associated with labeled landmarks while (b) depicts relevant structures pseudocolored for ease in visualization. The left kidney (K in (a)) has been reflected away from the left posterior abdominal wall to show the posterior aspect of its hilum. The two contributions to the ureter (yellow in (b)) can be observed emerging from the hilum; the superior contribution emerges posterior to the renal vein, and the inferior contribution descends from the renal hilum anterior to the renal vein. The left aberrant renal artery (pink in (b)) is observed branching before entering the substance of the kidney. The left ovarian artery (red in (b)) emerges from the anterior aspect of the aberrant left renal artery. Five segmental arteries and the renal vein are also observed entering the renal hilum (green and blue, respectively, in (b)).

During routine dissection, the hilum of the left kidney was cleaned and typical anatomical structures were identified, from anterior to posterior, as the renal vein, renal artery, and superior ureteral contribution (Figures 1 and 2). Following the removal of perirenal fat, a second ureteral contribution was found exiting the kidney, slightly inferior to the hilum. The two independent contributions, superior and inferior, descended 12.4 cm and 10.6 cm before they united anterior to the left psoas major muscle to descend over the left common iliac artery to the posterolateral aspect of the bladder (Figure 1).

Further dissection revealed an additional vascular structure, the left aberrant renal artery, entering the inferior pole of the left kidney (Figure 1). Cleaning the structure revealed its origin from the abdominal aorta 4 cm inferior to the left renal artery, which branched from the aorta below the superior mesenteric artery. The aberrant renal artery was then observed travelling in parallel with, but inferior to, the left renal vein and artery toward the left kidney. This artery was also observed passing anterior to the superior and inferior ureteral contributions and bifurcated just prior to reaching the parenchyma of the inferior renal pole of the left kidney. The left ovarian artery was observed branching from the aberrant renal artery nearly halfway to the left kidney. The left ovarian artery then descended medially, but adjacent to the superior ureteral contribution, and more inferiorly, adjacent to the united ureter to supply the left ovary (Figure 1).

## 3. Discussion

### 3.1. Left Bifid Ureter

The current case describes a left-sided bifid ureter that united anterior to the psoas major muscle before crossing the left common iliac artery and entering the posterolateral aspect of the bladder. During postmortem examination, duplicated ureters have been observed in 0.8% of cadavers studied [13]. This congenital malformation occurs during the development of the metanephric system which ascends from the sacral to the lumbar region to become the permanent kidney and ureter. The metanephric system is derived from two sources of intermediate mesoderm: the ureteric bud and metanephric blastema. Normally, the metanephric mesenchyme signals the ureteric bud to invade and branch into the mesenchyme, and the ureter reciprocally induces the mesenchyme to differentiate into nephrons [2]. The duplex system arises from a premature splitting of the ureteric bud or the development of two separate buds [14]. This anatomical variation is two times more commonly observed in females as compared to men, but the cause is currently unclear [6]. There have been reports that unilateral duplication is six times more common than bilateral duplication [6, 15]. It has also been reported that there is a one in eight chance for a child with a duplicated ureter to have a parent or sibling with a similar anomaly, thereby suggesting a heritable contribution to the above pathologies [15]. In general, reflux is the most common medical complication associated with a duplex kidney. Ureteroureteric reflux is most closely associated with incomplete duplication with an incidence of 22%, and vesicoureteric reflux is associated with complete duplication [16]. The respective incidences of these types of reflux are 69% and 72% [15, 16]. Other associated urinary tract complications include ureteropelvic junction obstruction and hydronephrosis [8].

### 3.2. Left Aberrant Renal Artery

Aberrant renal arteries occur in approximately 30% of the population, with left-side predominance, and are more commonly observed supplying the lower renal pole than the upper pole [17, 18]. The developmental mechanism of this variation is unclear, and the embryologic origin of the renal and adrenal arteries is under debate. The most widely held explanation is Felix's ladder theory [19]. This theory states that the blood supply to the developing mesonephros (including the adrenal glands, kidneys, and gonads) differentiates from the nine lateral mesonephric arteries (MAs). These vascular structures originate from the aorta and are divided into three groups: the first and second arteries are cranial, the third through fifth arteries are middle, and the sixth through ninth arteries are caudal. Normally, one middle lateral mesonephric (MLM) artery and one caudal lateral mesonephric artery persist and differentiate into the renal artery and gonadal artery, respectively, while the other vascular structures are obliterated [20]. However, the persistence of an additional MLM artery, for unknown reasons, results in the creation of either an accessory or aberrant renal artery, the latter of which is more commonly found entering the kidney at its lower pole [21].

Felix's ladder theory can be applied to developmental malrotation or improper positioning of the kidney to propose an alternative explanation for the left aberrant renal artery. During embryological development, the kidneys medially rotate and ascend to the lumbar region. It is during this ascent that the supplementary lateral mesonephric arteries should regress, but failure to do so could create aberrant renal arteries [22]. However, this is not likely to be the cause of this report's arterial variation because while reported cases did document multiple arterial supplies to the kidney, they also included a truncated ureter, renal arterial supply originating from various sources including the abdominal aorta and common iliac artery, and a pelvic kidney, which were not observed in the current case [23, 24].

In contrast to the ladder theory, Isogai et al. and Nobuyuki et al. recently proposed that the mesonephric arterial ladder is obliterated prior to the metanephric ascent and glomeruli development, meaning that the persistent renal artery is not derived from the mesonephric arterial system but originates from the aorta once the kidney is properly positioned in the posterior abdominal wall [19, 25]. Unlike Felix, these researchers maintain that the embryonic aorta has the potential to further branch after the MAs obliterate, but they also state that the definite renal artery may utilize the MA origins as its own. Nobuyuki et al. also found that the MAs, when observed during weeks 5-6 of embryological development, were segmental but not evenly distributed or symmetrical [25]. Perhaps the existence of more MA buds on the left lateral aspect of the aorta explains why aberrant renal arteries are more commonly found on the left.

The relationship of the ureters to the aberrant renal arteries is significant since the uncommon anatomy of the artery could present an additional risk factor during endoscopic surgery [26]. Damage or occlusion of an aberrant renal artery is known to lead to segmental infarcts and tissue necrosis, which could in turn damage a renal calyx and cause urine extravasation [27]. Vascular anomalies are also a common cause of congenital hydronephrosis, and obstructions at the UPJ can be due to an aberrant renal vessel crossing the ureter anteriorly [28, 29].

The present case report also addresses the overwhelming volume of vocabulary used to describe variations in the renal arterial supply. Gulas et al. have outlined the variable descriptions regarding additional renal arterial supply beginning with terms that began in the 16^th^ century until today [9]. Per their compilation, the terms “multiple” and “additional” were used most frequently when numerous renal arteries were present [9]. However, these terms lack specificity in that they do not explain the origins of the additional arteries nor how they enter the renal parenchyma. A classification system that accommodates variant renal segmental arteries as well as additional renal arterial supplies must be developed in order to efficiently and clearly share information regarding a patient's condition, thereby decreasing the risk of hemorrhage, segmental ischemia, and renovascular hypertension during renal transplant surgery [30].

### 3.3. Left Ovarian Artery

The final anomaly observed in this cadaver was the left ovarian artery originating from the left aberrant renal artery. The ovarian artery typically arises anteromedially from the abdominal aorta several centimeters below the renal arteries, while an ovarian artery arising from the renal, lumbar, adrenal, or iliac arteries occurs at a rate of 10-20% [31]. In order to understand why the ovarian artery originates from the aberrant renal artery, it is prudent to first explore the embryological development of the ovary itself.

The gonad develops from the urogenital fold, a structure superior to the developing kidney. As the gonads descend within the elongating body, the ascending kidneys and the aortic mesonephric blood supply pass posterior to the gonads, causing the gonadal vascular supply to also originate from the lateral mesonephric arteries. The lateral mesonephric branches that potentially reach to supply the descending gonads are categorized in one of two ways: the first type is cranial to the renal hilum and the second is caudal to it. Only one of the lateral mesonephric arteries will persist, thereby becoming the gonadal artery. The relationship of the gonadal artery, or in this case the ovarian artery, to the hilum of the kidney paired with the kidney's ascension level (which is higher on the left) will predict the gonadal artery's relationship to neighboring structures, including the renal vein, renal artery, and aorta [32]. While rare, gonadal arteries have been observed to originate from numerous sources due to the anastomoses between the persistent mesonephric arteries as the mesonephric system develops. These sources include the abdominal aorta, renal artery, or accessory renal artery and the variations are often left-sided [32, 33]. The path of this left ovarian artery is consistent with that of an ovarian artery originating from the aorta, so it is unlikely that this left ovarian artery would experience compression. A more likely complication of origination of the ovarian artery from the aberrant renal is an ischemic event due to the reduced caliber of the aberrant artery and the ovarian artery. This ischemia may then lead to infarction or ischemic reperfusion injury to the ovary [34].

## 4. Conclusion

Postmortem urogenital and vascular anomalies were observed in a 77-year-old female cadaver. No reports were found in the literature documenting the combination of variants observed: left bifid ureter, left aberrant renal artery, and left ovarian artery originating from the aberrant vessel. These findings are clinically important as the aberrant artery reinforces the notion that multiple renal arterial sources are more prevalent than previously believed, meaning that new imaging modalities such as endoluminal ultrasonography, computed tomography angiography (CTA), and magnetic resonance angiogram (MRA) could replace the older methods such as intravenous urography and angiography. Lastly, the acceptance and utilization of a common nomenclature will facilitate the sharing of information between academic, surgical, and radiological fields of study. The aberrant renal vessel can also cause hydronephrosis, which, along with reflux, UPJO, and segmental infarction of the kidney, can manifest itself as a result of a duplex renal system. The knowledge of these potential anatomical variations must be paired with an understanding of their embryological origins and imaging techniques to aid proper clinical diagnosis and treatment.
